# Phloretic acid requires the insulin/IGF-1 pathway and autophagy to enhance stress resistance and extend the lifespan of *Caenorhabditis elegans*

**DOI:** 10.3389/fphar.2024.1384227

**Published:** 2024-03-27

**Authors:** Bo Li, Li Dong, Wei Meng, Shi-Ying Xiong, Gui-Sheng Wu, Wen-Zhe Ma, Huai-Rong Luo

**Affiliations:** ^1^ State Key Laboratory of Quality Research in Chinese Medicine, Macau University of Science and Technology, Macau, China; ^2^ The Affiliated Traditional Chinese Medicine Hospital, Luzhou, China; ^3^ Key Laboratory of Luzhou City for Aging Medicine, Department of Pharmacology, School of Pharmacy, Southwest Medical University, Luzhou, China; ^4^ Central Nervous System Drug Key Laboratory of Sichuan Province, Luzhou, China

**Keywords:** Phloretic acid, *Caenorhabditis elegans*, anti-aging, stress response, autophagy

## Abstract

**Objective:** In humans, aging is associated with increased susceptibility to most age-related diseases. Phloretic acid (PA), a naturally occurring compound found in Ginkgo biloba and Asparagus, exhibits has potential as an anti-aging agent and possesses antioxidant, anti-inflammatory, and immunomodulatory properties. This study aimed to investigate the effects of PA on longevity and stress resistance in *Caenorhabditis elegans* (*C.elegans*) and the mechanisms that underlie its effects.

**Methods:** First, we examined the effects of PA on lifespan and healthspan assay, stress resistance and oxidative analysis, lipofuscin levels. Second, we examined the insulin/insulin-like pathway, mitochondria, autophagy-related proteins, and gene expression to explain the possible mechanism of PA prolonging lifespan.

**Results:** Our findings demonstrated that PA dose-dependently extended the *C.elegans* lifespan, with 200 μM PA showing the greatest effect and increased the *C.elegans* lifespan by approximately 16.7%. PA enhanced motility and the pharyngeal pumping rate in senescent *C.elegans* while reducing the accumulation of aging pigments. Further investigations revealed that daf-16, skn-1, and hsf-1 were required for mediating the lifespan extension effect of PA in *C.elegans* since its impact was suppressed in mutant strains lacking these genes. This suggests that PA activates these genes, leading to the upregulation of downstream genes involved in stress response and senescence regulation pathways. Furthermore, PA did not extend the lifespan of the RNAi *atg-18* and RNAi *bec-1* but it attenuated SQST-1 accumulation, augmented autophagosome expression, upregulated autophagy-related gene expression, and downregulated S6K protein levels. These findings suggest that the potential life-extending effect of PA also involves the modulation of the autophagy pathway.

**Conclusion:** These findings results highlight the promising anti-aging effects of PA and warrant further investigation into its pharmacological mechanism and medicinal development prospects.

## 1 Introduction

Aging poses significant international concerns, bringing forth formidable socioeconomic and healthcare challenges for both developed and developing countriesIt represents an irreversible progression accompanied by process, physiological dysfunction (Partridge et al., 2018).

Aging is associated with an increased incidence of age-related diseases such as cancer, diabetes, hypertension, Alzheimer’s disease, and Parkinson’s disease, leading to a decline in quality of life and increased healthcare expenses. Consequently, there is an imperative need to understand the underlying molecular mechanisms of aging and explore strategies for retarding or reversing the onset of age-related diseases. Notably, natural compounds serve as valuable resources for the development of anti-aging herbal medicinesand their usehas garnered significant attention through external interventions, due to their potential in controlling the progression of age-related diseases and elucidating their molecular mechanisms.

Phloretic acid (PA) also known as desaminotyrosine, hydro-p-coumaric acid, Phloretate, and 3-(4-hydroxyphenyl)propanoic acid, is a naturally occurring phenolic compound that can be produced by the hydrogenation of p-coumaric acid or synthesized from phloretin, a byproduct of apple tree. This compound finds wide application as an intermediate in pharmaceutical formulations, such as esmolol hydrochloride, which exhibits beta-adrenergic receptor antagonist activity and specifically targets the heart. Recent studies have demonstrated the potential of topically applying this drug as a novel therapeutic approach for diabetic foot ulcers (Kulkarni et al., 2022; Rastogi et al., 2023a; Rastogi et al., 2023b). Another commonly used medication is cetraxate hydrochloride, which acts as a mucosal blood flow enhancer by improving microcirculation in the gastric mucosa. It enhances mucosal resistance and stimulates PGE2 and PGI2 production in the gastric mucosa. Additionally, it inhibits gastric acid secretion and pepsin activation while promoting ulcer healing (Suzuki et al., 1976; Ishimori et al., 1979). Numerous experiments have demonstrated that PA, which serves as an intermediate product in pharmaceuticals, has various effects, including anti-inflammatory, antioxidant, and immunomodulatory effects; however, its anti-aging properties have not been studied. Therefore, further are currently available. Further research exploring this aspect is warranted.

*C.elegans* is considered the only classic multicellular model organism for aging and neurodegenerative research (Wong et al., 2020). More than half a century ago, Sydney Brenner first introduced *C. elegans* as an experimental model organism that has a short life cycle, simple physiological structure, a large number of offspring, is easily reproduced, and shares high genetic homology with mammals to be manipulated without any difficulty (Brenner, 1974). Therefore, *C.elegans* has become an ideal model for aging research and an important model for anti-aging drug screening to clarify the potential effects of bioactive compounds on health and lifespan.Our previous investigation into the screening of natural drug compounds using Cryptobacterium hidradii as a model organism yielded intriguing findings (Shi et al., 2023). In this study, we chose *C.elegans* to study the anti-aging and anti-stress effects of PA.

## 2 Materials and methods

### 2.1 Chemicals

PA (purity≥98%) was purchased from Shanghai Yuanye Bio-Technology Co. Ltd. (Shanghai, China), 5-fluorodeoxyuridine (FUDR), and 5-(and-6)-chloromethyl-2′,7′-dichlorodihydrofluorescein diacetate (CM-H2DCFDA), and ethanol from Sigma-Aldrich (United States).

### 2.2 Worm strains and maintenance

All worm strains used in this study were provided by the *Caenorhabditis* Genetic Center (CGC; University of Minnesota, Minneapolis, MN): N2 (wild-type), CF1038 daf-16 (mu86) I., EU1 skn-1 (zu67)IV., PS3551 hsf-1 (sy441)I., RB754 aak-2 (ok524)X., RB759 akt-1 (ok525)V., VC204 akt-2 (ok393)X., CB4876 clk-1 (e2519)III., CF1553 [(pAD76) sod-3:GFP + rol6 (su1006)], CL2166 dvIs19 [pAF15 (gst-4:GFP::NLS)], SJ4100 (zcIs13 [hsp-6:GFP]), SJ4005 zcIs4V (hsp-4:gfp), SJ4058 zcIs9 [hsp60:GFP + lin-15 (+)], NL5901 [unc54p: alphasynuclein:YFP + unc-119 (+)], DA2123 [lgg-1p:GFP:lgg-1 + rol6 (su1006)], TJ356 [daf-16p:daf-16a/b:GFP + rol-6] and others. Worms were cultured on nematodegrowth media (NGM) at 20°C, except for specific strains requiring alternative conditions.

### 2.3 Lifespan assay

All strains were cultured on fresh NGM plates for 2–3 generations without starvation. When synchronized larvae reached the L4 stage, worms were transferred to an NGM plate containing PA and FUDR (50 mg/mL, to inhibit nematode reproduction). Dead worms were counted dailywith worms responding to slight touch from the worm pickerrecorded as alive. Worms that were missing, dried or hatched internally were censored from the lifespan count. Experiments were performed with at least 60 nematodes in each group.

### 2.4 Phenotype analysis

Lipofuscin analysis: On the 5th and 10th days of PA treatment, the worms were collected and photographed using a fluorescence microscope (Leica DFC 7000T) at an excitation wavelength of 360–370 nm and an emission wavelength of 420–460 nm to quantifylipofuscin accumulation. At least 30 worms were included in each group, and images of the nematodes were processed using ImageJ.

Body-bending experiment: The worms were synchronized and incubated overnight at 20°C; L1-stage larvae were then incubated on NGM plates until late L4stage.On days 5 and 10, the wormswere transferred to experimental platesadded to water droplets to stabilize for 1 min, and subjected to body-bending analysis under a microscope within 20 s. A minimum of 30 worms were included in each group.

Pharyngeal pumping rate: Following the same preliminary treatment as the body-bending test, the number of pharyngeal pumping events was recorded under a microscope on the 5th and 10th days for the 20 s. A minimum of 30 worms were included in each group.

Mobility assay: This was carried out on days 9, 14, and 18. Sixty worms were observed, and the locomotion of motion A to motion C was quantitatively measured according to previous protocols (Wang H. et al., 2018). All experiments were performed in triplicate.

### 2.5 Stress resistance assays

For the heat shock assay, worms were treated with PA for 7 days from the L4 larvae stage, then transferred to a new NGM plate and incubated at 35°C forheat shock. Dead worms were counted hourly until all worms succumbed. In the oxidative stress assay, worms were treated with PA as described above, then transferred to NGM plates containing paraquat (20 mM, Sigma‒Aldrich). Dead of worms were counted daily. Each group comprised at least 60 nematodes, and the experiment was repeated threetimes.

### 2.6 Reactive oxygen species assay

L4 stage worms were spread on experimental NGM plates containing PA, paraquat (20 mM), or N-acetyl-L-cysteine (1 mM) and incubated at 20°C for 6 days. Afterward, the worms were collected, washed with M9 buffer at least three times, stained with an H2DCF-DA (50 μM) probe and shaken at 35°C for 60 min according to the ROS detection kit (Hui et al., 2020).ROS determination was performed by imaging under a fluorescence microscope. The experiment was independently repeated at least three times, with each experiment involving at least 30 worms.

### 2.7 DAF-16:GFP translocation assay

TJ356 worms cultured to the L4 stage were transferred to the experimental group (200 μM PA)or two control groups.An NGM plate containing TJ356 and OP50 bacteria was subjected to heat shock (37°C, 15 min) as a positive control, while another control plate was placed in a 20°C incubator as a negative control. The localization of DAF-16:GFP was observed every hour under a fluorescence microscope. The green fluorescent nuclear aggregation particles in the TJ356 worms served as the index of the DAF-16 gene in the nucleus (Wang et al., 2021).

### 2.8 Oil red-O staining

Following synchronization, the Subsequently were spread onto experimental plates and grown until adulthood. Approximately 1000 worms from both the experimental and control groups were collected, subjected to multiple washes with PBS to eliminate excess bacterial fluid, and treated with Nile red staining reagent. After fixation with paraformaldehyde (4%) for 25 min, the worms underwent by two washes in PBS+1% Triton buffer solution. Subsequently, Nile red reagent (5 mg/mL) was added under light-protected conditions; after an incubation period of 2 min, the worms were washed three or more times with PBS+1% Triton buffer solution before microscopic examination (Leica DFC7000T). ImageJ software was used for image analysis. Each experiment was repeated three times, utilizing at least 30 worms per repetition.

### 2.9 RNA interference

RNA interference (RNAi) was performed as previously described (Shi et al., 2023). RNAi was conducted by feeding HT115 (DE3) (Fire Lab) bacteria vectors L4440 (control), atg-18, and bec-1, which produce dsRNA against the target gene. The RNAi worm lifespan assay was conducted according to previous methods (Beifuss and Gumienny, 2012).

### 2.10 Autophagy assay

To detect autophagy in nematodes, the number of GFP-positive foci on the lgg-1 autophagic vesicles was used to evaluate autophagy. DA2123 worms showed diffuse fluorescence in the cytoplasm of various tissues. Through the appearance of fluorescent points, the formation of autophagosome structures can be observed and quantified.

### 2.11 Real-time quantitative PCR Assay

Approximately 3,000 synchronized N2 worms were cultured to late the L4 or early adult stage, transferred to the experimental group (with or without 200 μM PA, containing 20 mM FUDR) and cultured at 20°C for 24 h. Total RNA was extracted using the Steadypure Universal RNA Extraction Kit (Accurate Biology) and reverse-transcribed into cDNA using the PrimeScript™RT reagent kit with gDNA Eraser (Perfect Real Time). mRNA expression was quantified by the SYBR Green Premix Pro Taq HS qPCR Kit (Rox Plus) on the QuantStudio 6 Flex system. The relative mRNA expression levels of genes were calculated using the 2^−ΔΔCT^ method and normalized to the expression of the gene cdc-42 (Yanase, 2020).

### 2.12 Western blot

The worms were collected on day 6 of the PA intervention. Protein was extracted by homogenization using a sonicator, and protein concentrations were determined by a Bicinchoninic Acid Protein Assay Kit (Beyotime). The experiment was conducted according to the previous protocol (Jeong et al., 2018). ImageJ software was used for image analysis.

### 2.13 Statistical analysis

Statistical analyses were conducted using SPSS 26.0 Statistics and GraphPad Prism 7.0 software. Fluorescence quantification, oil red quantification, and protein quantification statistics were performed using ImageJ 1.8.0. Lifespan experiments were analyzed using Kaplan-Meier survival analysis. Other data were expressed as the mean ± SD, unless otherwise stated. The *p* values were determined by two-tailed *t*-test.A *p*-value <0.05 was considered a significant difference.

## 3 Results

### 3.1 PA can extend the lifespan of *C. elegans* and improve aging-related phenotypes

**FIGURE 1:**
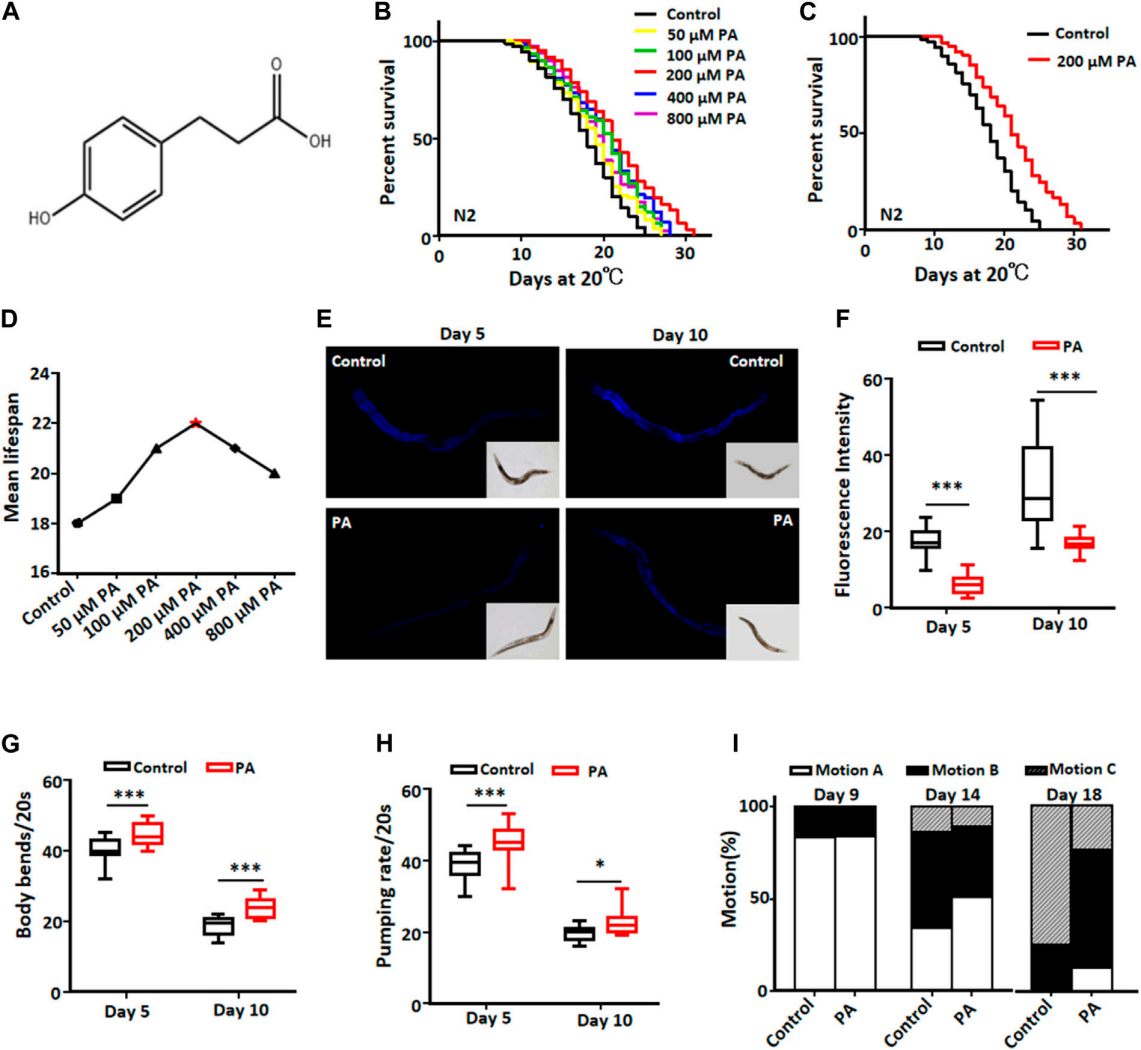
PA extends the lifespan of *C. elegans* and enhances its health. **(A)** The chemical structure of PA is depicted. **(B)** Survival curves of wild-type N2 worms at 20°C with or without different concentrations (50, 100, 200, 400, and 800 µM) of PA are shown. **(C)** Survival curves of wild-type N2 worms treated with or without 200 μM PA at 20°C are presented; statistical analysis indicated significant differences between the groups (*p* < 0.001, log-rank test). **(D)** The mean lifespan of N2 nematodes was measured after treatment with various concentrations of PA. **(E,F)** The lipofuscin content in nematodes treated with a concentration of 200 μM PA; the relative fluorescence intensity was calculated using ImageJ software and is presented. Statistical analysis revealed significant differences between the groups (mean ± SD, n ≥ 30; ***p < 0.001; *t*-test). **(G,H)** Quantification results for body bends and pharyngeal pump times in wild-type N2 worms are provided; statistical analysis shows statistically significant differences (mean ± SD, n ≥ 30; *p < 0.05 and ***p < 0.001; *t*-test). **(I)** Movement patterns observed in wild-type N2 worms with or without a concentration of 200 μM PA at a temperature of 20°C are categorized into three types: motion A represents spontaneous activity; motion B corresponds to physical movement after prodding by metal wire stimulation; and motion C denotes only simple head or tail movement in response to stimulation.

To investigate the impact of PA on lifespan, we treated wild-type N2 worms with various concentrations of PA (Figure 1A). Our results showed that PA at each concentration could prolong the lifespan of *C. elegans* to a certain extent, while compared with the control, 200 μM PA had the greatest effect and increased the lifespan of *C. elegans* by 16.7% (Figures 1B–D). Lipofuscin is a yellow‒brown pigment that accumulates with age (Sitte et al., 2000). PA significantly decreased lipofuscin deposition (Figures 1E,F) and improved the movement ability and pharyngeal pump activity of *C. elegans* (Figures 1G–I) on days 5 and 10 of adulthood. These results indicated that PA could promote the health of *C. elegans*.

### 3.2 The transcription factor FOXO/DAF-16 is required for PA to extend the lifespan of *C. elegans*

**FIGURE 2:**
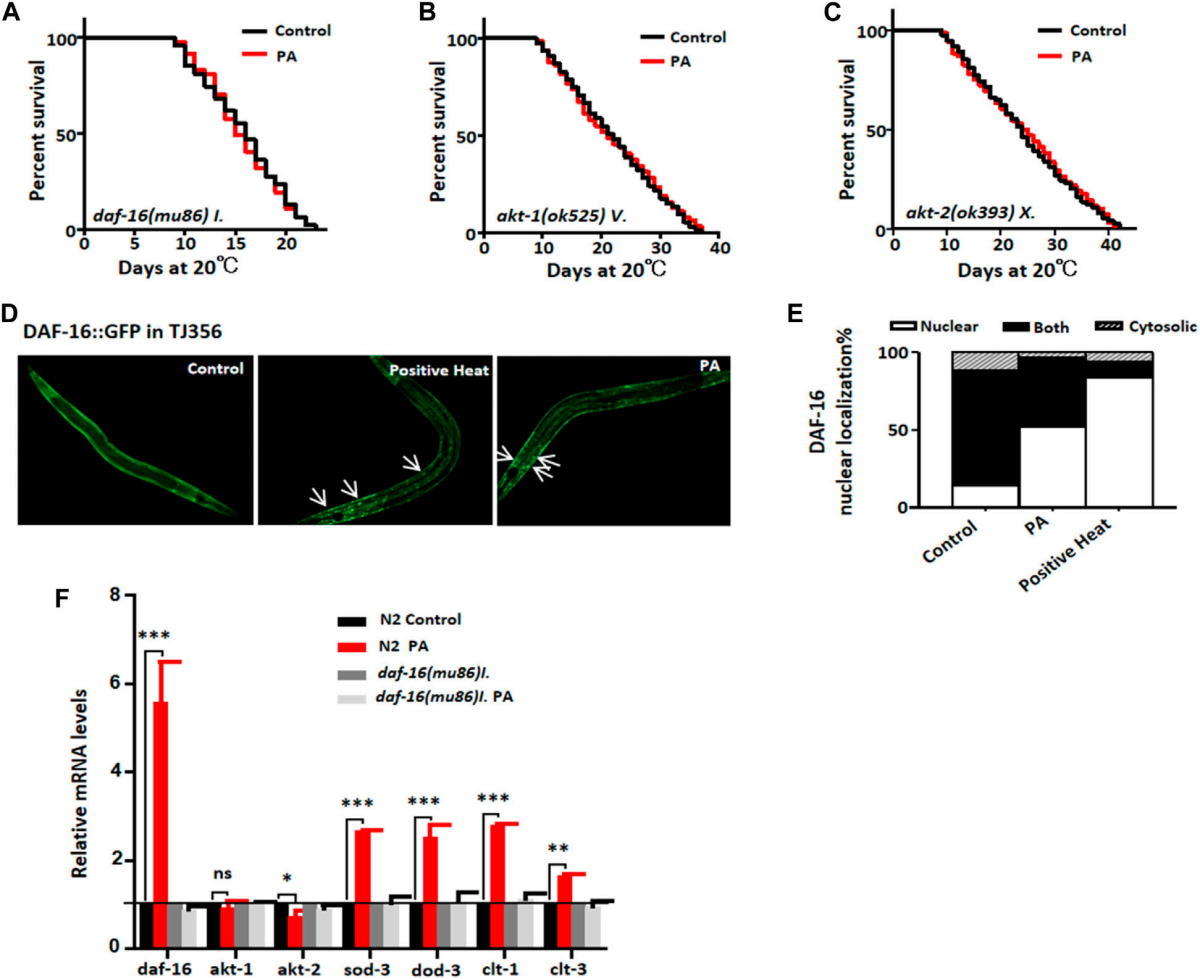
PA prolonged the lifespan of *C. elegans* and required *FOXO/DAF-16*. **(A–C)** Survival curves of *daf-16* (*mu86)I.*, *akt-1* (*ok525)v*., and *akt-2 (ok393)X* (mean ± SD, n ≥ 3; *t*-test). **(D,E)** Representative images and quantification demonstrating the effect of 200 μM PA on *daf-16* nuclear localization in the TJ356 strain. **(F)** Relative expression levels of downstream genes regulated by *daf-16* in the wild-type N2 L4 stage and mutant CF1038 treated with 200 μM PA for 24 h (mean ± SD, n ≥ 3; *p < 0.05, **p < 0.01, ***p < 0.001; NS, not significant; *t*-test).

DAF-16, a nematode homolog of the mammalian FOXO transcription factor, plays a crucial role in coordinating diverse biological processes, including stress tolerance, development, reproduction, lipid storage, and longevity (Kim and Webb, 2017). In the insulin signaling pathway, the AKT-1 and AKT-2 kinases act upstream of DAF-16 (Hesp et al., 2015). Our findings demonstrated that PA failed to extend the lifespan of daf-16 (mu86) loss-of-function mutant worms (Figure 2A)and did not significantly prolong the lifespan of Akt-1 or Akt-2 kinase-deficient mutants (Figures 2B,C). Notably, PA enhanced the nuclear accumulation of the DAF-16:GFP fusion protein (Figures 2D,E), and upregulates the mRNA levels of the DAF-16/FOXO downstream target genes sod-3, dod-3, clt-1, and clt-3. However, in daf-16 (mu86) loss-of-function mutants, the expression levels of these genes remained unaltered regardless of the presence or absence of PA (Figure 2F). Collectively, these results suggest that the activation of FOXO/DAF-16 is required for the PA-mediated extension of the *C. elegans* lifespan.

### 3.3 Ability of PA to prolong the lifespan of *C. elegans* depends on HSF-1

**FIGURE 3:**
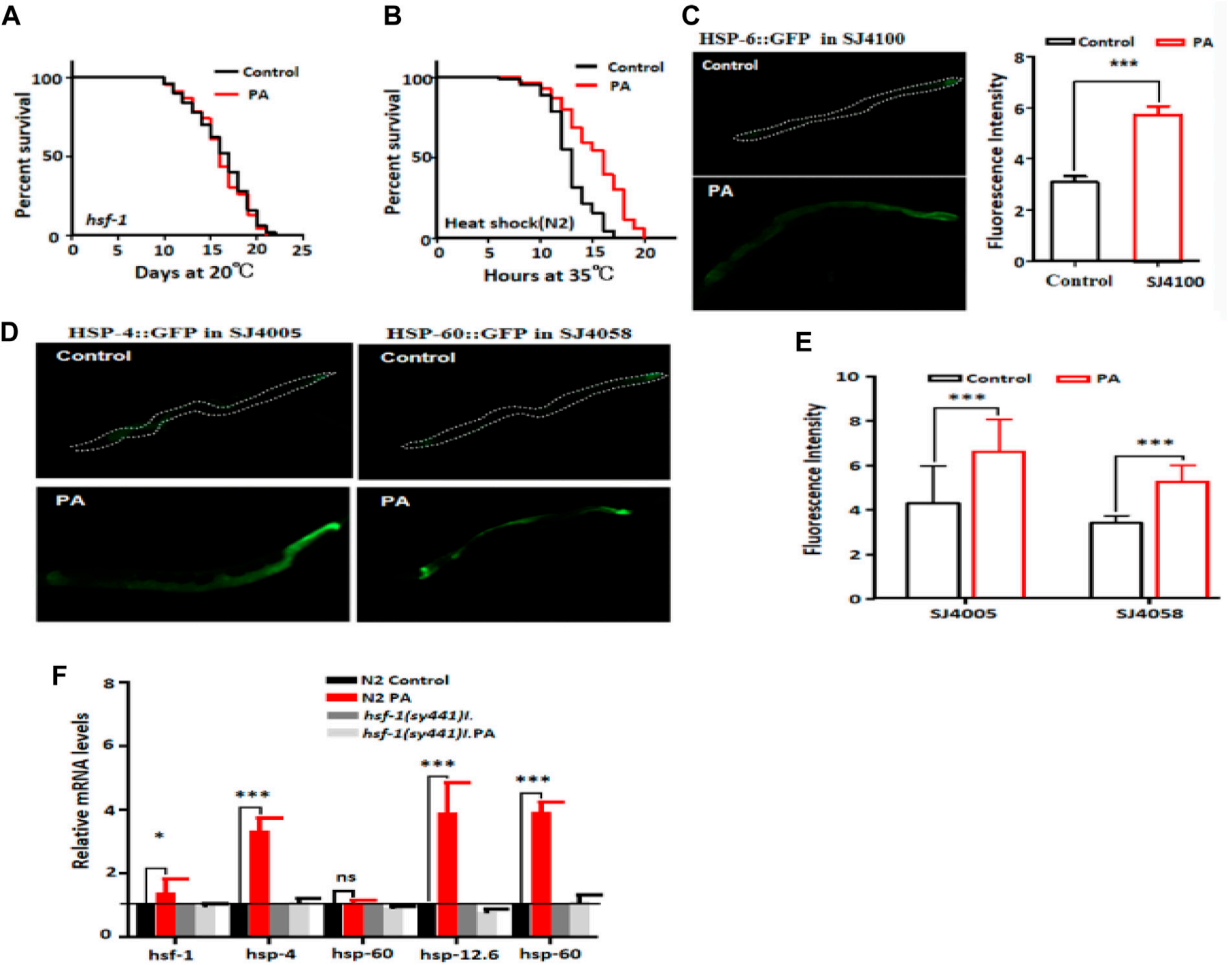
PA administration extends lifespan in an HSF-1-dependent manner. **(A)** Survival curve of patients treated with mutant PS3551 with or without 200 μM PA (mean ± SD, n ≥ 3; log-rank test). **(B)** The survival rate of wild-type N2 plants treated with or without 200 μM PA at 35°C significantly increased by 33.9% (*p* < 0.001, log-rank test). **(C–E)** Quantification of fluorescence intensity upon treatment with 200 μM PA in the worm strains SJ4100 (*hsp-6p::GFP*), SJ4005 (*hsp-4::GFP*), and SJ4058 (*hsp-60::GFP*) revealed significant upregulation compared with the control conditions (mean ± SD, n ≥ 3; ***p < 0.001; *t*-test). **(F)** The relative expression of downstream genes of *hsf-1* after L4 stage wild-type N2 and the *hsf-1* mutant were treated with 200 μM PA for 24 h (mean ± SD, n ≥ 3; ns, not significant; ***p < 0.001; log-rank test).

Heat shock transcription factor (HSF-1) regulates the expression of heat-induced target genes, including small heat shock proteins, and plays a crucial role in longevity regulation and protein toxicity management. It serves as a key downstream transcription factor of the insulin signaling pathway (Brunquell et al., 2017). Our investigation aimed to determine whether hsf-1 also plays a critical role in lifespan regulation within the context of PA treatment targeting the insulin signaling pathway. First, we examined the impact of PA on the lifespan of the HSF-1 mutant PS3551. Our results indicated that PA did not extend the lifespan of this mutant (Figure 3A). Next, we conducted a heat shock experiment on wild-type N2 nematodes and found that PA-treated nematodes exhibited longer survival times then control worms at 35°C (Figure 3B). This suggests that PA confers a certain level of resistance to heat stress. HSP-6 is involved in regulating misfolded protein binding activity in nematodes and participates in mitochondrial unfolded protein responses during heat shock (Govindan et al., 2019). Subsequently, we investigated fluorescence expression using the SJ4005 (hsp-4:GFP), SJ4058 (hsp-60:GFP), and SJ4100 (hsp-6p:GFP) strains to assess the effect of PA treatment. Our results demonstrated an increase in protein expression after PA treatment (Figures 3C–E). Moreover, significant increases in the mRNA levels of the downstream target genes *hsp-1*, *hsp-4*, *hsp-6*, and *hsp-60* were detected following PA treatment (Figure 3F). These findings suggest that by inhibiting the IIS signaling pathway, PA can activate downstream mechanisms to regulate longevity through HSF-1 in *C. elegans.*

### 3.4 PA improved stress resistance and extended the lifespan of *C. elegans* by activating SKN-1

**FIGURE 4:**
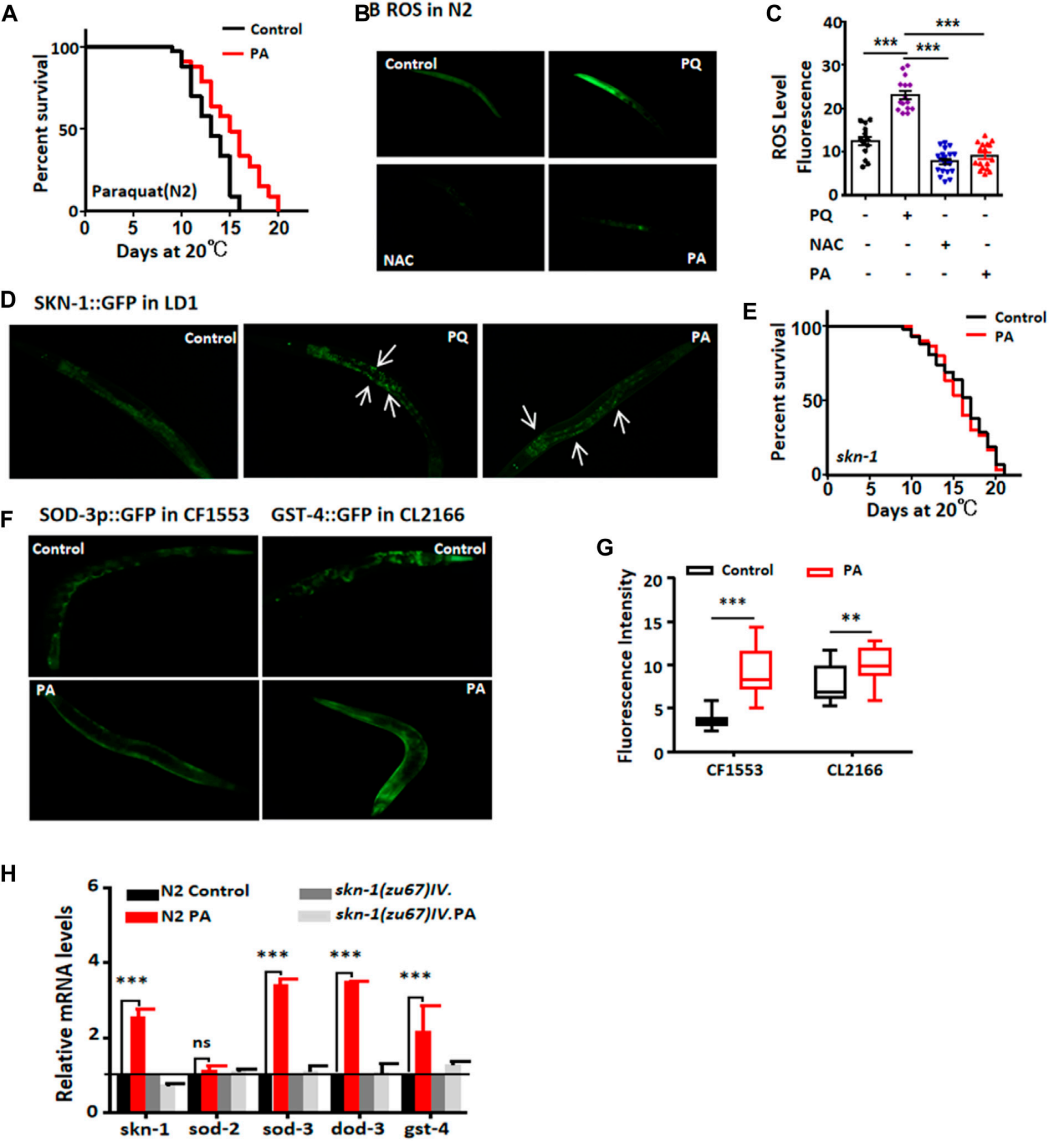
PA significantly enhances the antioxidant capacity and extends the lifespan of *C. elegans* in an *skn-1* expression-dependent manner. **(A)** Lifespan analysis was conducted on wild-type N2 worms exposed to paraquat for 6 days with or without PA treatment (mean ± SD, n ≥ 30; *t*-test). **(B,C)** Quantitative analysis of representative images of ROS levels in N2 nematodes treated with or without 200 μM PA (±SD, n ≥ 30; ***p < 0.001; *t*-test). **(D)** Representative images of LD1 nucleation upon treatment with 200 μM PA. **(E)** Lifespan analysis was carried out on *skn-1* mutant worms treated with or without PA. **(F,G)** Quantitative assessment of the fluorescence intensity of CF1553 (*SOD-3p::GFP*) and CL2166 (*GST-4::GFP*) with or without 200 μM PA on day 7 (mean ± SD, n ≥ 30; **p < 0.01 and ***p < 0.001; *t*-test). **(H)** Relative expression levels of downstream genes regulated by *skn-1* after treatment with or without PA (mean ± SD, n ≥ 3; ***p < 0.001; ns, not significant; *t*-test).

Excessive accumulation of free radicals leads to tissue damage and degenerative changes in organs (Blackwell et al., 2015). Our findings demonstrate that PA treatment enhances the survival rate of worms exposed to paraquat and reduces ROS levels (Figures 4A–C). The transcription factor SKN-1/Nrf-2 regulates the expression of numerous antioxidant enzymes and phase I detoxification enzymes. We observed a significant increase in fluorescence intensity in the LD1 mutant strain treated with PA compared with the control group, albeit slightly weaker than that in PQ-treated worms (positive control Figure 4D). However, PA failed to extend the lifespan of *skn-1* (zu67) mutants (Figure 4E). SOD and GST-4 play crucial roles in the oxidative stress response; maintaining high levels of SOD-3 and GST-4 expression may contribute to delaying aging (Pohl et al., 2019). Therefore, we treated the sod-3:gfp (CF1553) and gst-4:gfp (CL2166) mutant strains with PA for 7 days, followed by exposure to heat stress for 2 h. Remarkably, PA treatment significantly increased the protein expression of SOD-3 and GST-4 (Figures 4F,G). Additionally, the mRNA levels of *skn-1* and its downstream genes *skn-1*, *sod-3*, *dod-3*, and *gst-4* in N2 nematodes treated with PA were elevated; however, no alterations were detected in their expression within the skn-1 mutant strain (Figure 4H).

### 3.5 PA extends the lifespan of *C. elegans* through the mitochondrial signaling pathway and is implicated in lipid metabolism

**FIGURE 5:**
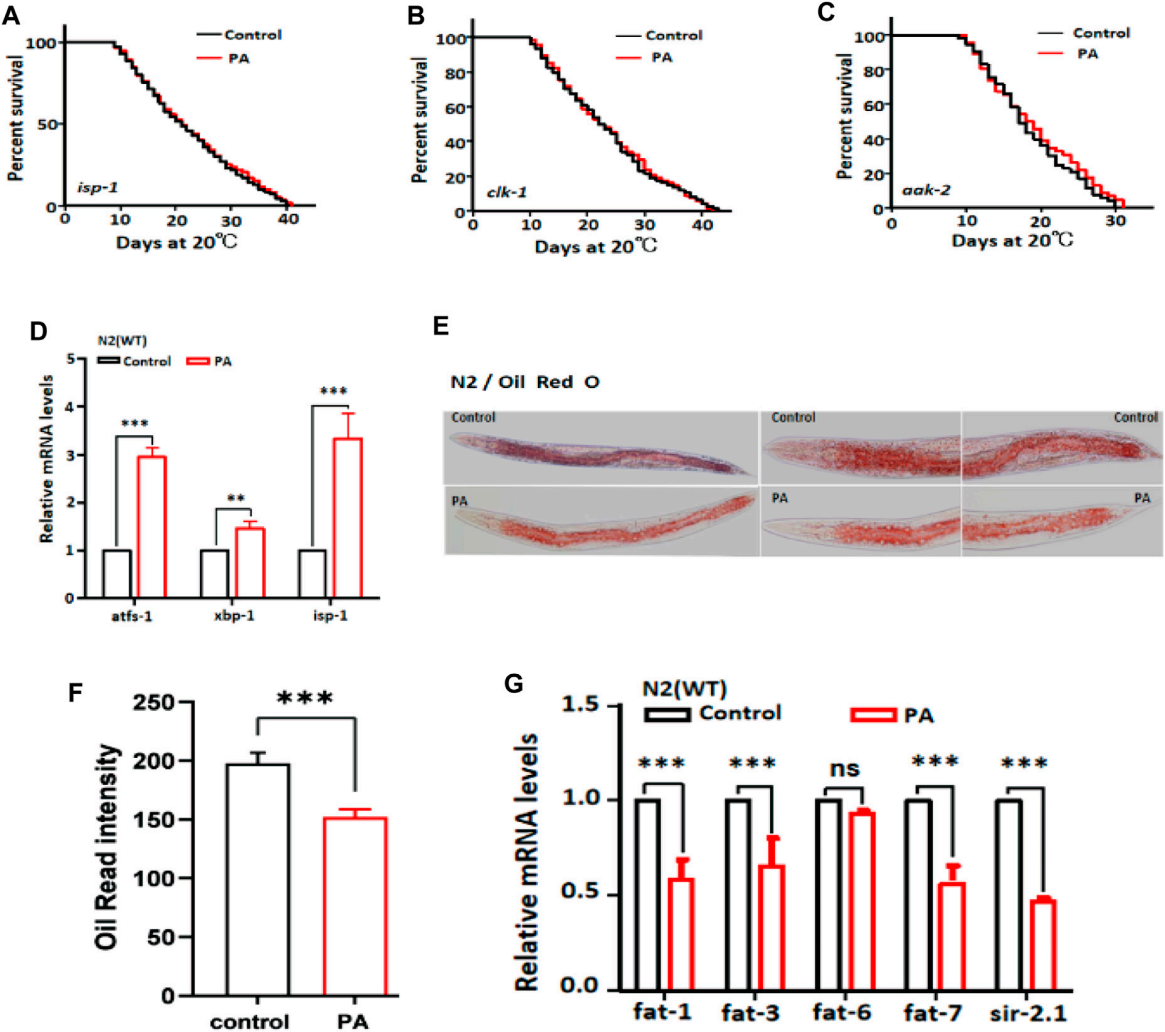
PA extends the lifespan of *C. elegans* and modulates lipid metabolism via the mitochondrial pathway. **(A–C**) Survival curves of the mitochondrial dysfunction mutants *isp-1*, *clk-1*, and *aak-2* treated with 200 μM PA (mean ± SD, n ≥ 3; log-rank test). **(D)** Relative expression levels of mitochondrial genes in N2 worms after 24 h of treatment with 200 μM PA (mean ± SD, n ≥ 3; **p < 0.01 and ***p < 0.001; *t*-test). **(E,F)** Effects of wild-type N2 on fat content analyzed with or without 200 μM PA (mean ± SD, n ≥ 30; **p < 0.01; *t*-test). **(G)** mRNA expression levels related to fat metabolism genes were assessed after 24 h of treatment with 200 μM PA (mean ± SD, n ≥ 3; ***p < 0.001; ns, not significant; *t*-test).

As previously discussed, the longevity of *C. elegans* is attributed to the antioxidant activity of PA. Mitochondrial dysfunction can lead to oxidative stress and impaired mitochondrial performance due to ROS production (Dilberger et al., 2019). We investigated whether mitochondrial function plays a pivotal role in the lifespan extension induced by PA. Our findings demonstrated that PA fails to extend the lifespan of mutants with mitochondrial dysfunction, namely, *isp-1*, *clk-1*, and *aak-2* (Figure 5A–C). This finding suggested that PA modulates mitochondrial function in *C. elegans*. Furthermore, we observed upregulation of the mRNA levels of the mitochondria-associated transcription factors *atfs-1*, *xbp-1*, and *isp-1* following PA treatment in *C. elegans* (Figure 5D). Antioxidants have been reported to enhance lipid metabolism (Amorim et al., 2022). Our results indicate that PA has the potential to attenuate Oil Red O staining intensity as an indicator of lipid storage and downregulate the mRNA expression of the target genes *fat-1*, *fat-3*, *fat-7*, and *sir-2.1, which are* associated with lipid metabolism (Figures 5E–G).

### 3.6 PA can prolong the lifespan of *C. elegans* via autophagy

**FIGURE 6:**
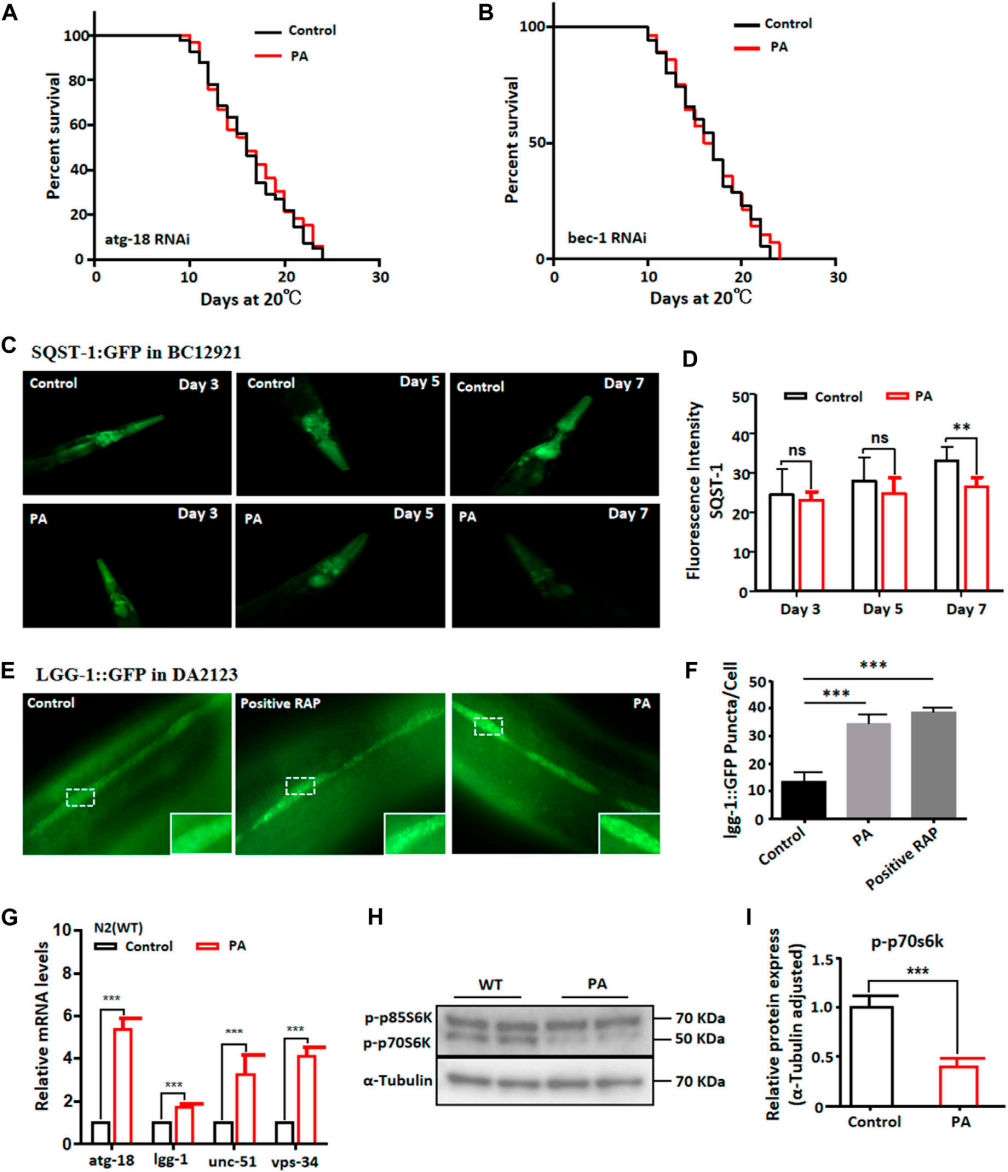
PA extended the lifespan of *C. elegans* through the autophagy pathway. **(A,B)** Survival curves of mutant RNAi *atg-18* and RNAi *bec-1* with or without 200 μM PA (mean ± SD, n ≥ 30; log-rank test). **(C,D)** Representative images and quantification of *BC12921* (SQST-1) treated with PA for 3, 5, and 7 (mean ± SD, n ≥ 30; **p < 0.01; ns, not significant; *t*-test). **(E,F)** Representative images and quantification of DA2123 (*lgg-1*) treated with 200 μM PA for 48 h (mean ± SD, n ≥ 30; ***p < 0.001; *t*-test). **(G)** Relative expression analysis of autophagy-related genes after treatment with 200 μM PA for 24 h (mean ± SD, n ≥ 3; ***p < 0.001; *t*-test). **(H,I)** S6K protein expression remained unchanged following PA treatment (mean ± SD, n ≥ 3; ***p < 0.001; log-rank test).

Autophagy is a highly conserved degradation process that, when triggered by stressful conditions, eliminates damaged intracellular macromolecules to maintain cellular homeostasis and promote organismal health and development (Palmisano and Meléndez, 2019). Recent studies have established links between autophagy and various human diseases. Interestingly, many interventions that extend lifespan increase the accumulation of autophagosomes (Peña-Ramos et al., 2022). We examined the fluorescence of BC12921 following PA treatment and evaluated the lifespans of atg-18 and bec-1 mutant nematodes associated with autophagy genes. RNAi targeting autophagy genes, including *atg-18* and *bec-1*, did not definitively prolong lifespan (Figures 6A,B). However, we observed that PA treatment reduced the degradation of the phagocytic substrate SQST-1 in BC12921 (Figures 6C,D) while increasing the fluorescence intensity of the autophagosome marker lgg-1 in DA2123 (Figures 6E,F). These findings suggest that PA can enhance autophagic activity. Furthermore, the mRNA transcription levels of *atg-18*, *lgg-1*, *unc-51*, and *vps34* were significantly upregulated upon PA treatment (Figure 6G). Intriguingly, the protein level of S6K, a crucial downstream gene regulated by the mTOR signaling pathway, decreased significantly (Figures 6H,I). This finding implies that the lifespan extension induced by PA may be attributed to enhanced autophagy through activation of the mTOR pathway.

## 4 Discussion

Aging is a progressive and time-dependent process (Fulop et al., 2023), closely associated with the development of most chronic diseases and increased morbidity and mortality (Roy et al., 2023). The pursuit of effective strategies to enhance a healthy lifespan has long been a fundamental objective in aging research. Natural compounds derived from herbal medicine have garnered significant attention; therefore, this study selected PA, a natural compound found in sources such as Ginkgo biloba and Asparagus, as the subject of investigation to elucidate its phenotypic effects and potential underlying mechanisms for extending nematode lifespan. In our study, we observed that different concentrations of PA significantly increased the lifespan of *C. elegans*. Notably, 200 μM PA exhibited the most prominent effect. Furthermore, we investigated whether PA could enhance the healthy lifespan of *C. elegans* and found that 200 μM PA improved body bending, the pharyngeal pumping rate, motility, and reduced lipofuscin deposition in these nematodes. As enhanced stress resistance was also reflected in the longevity phenotypic characteristics of *C. elegans*, we further examined the stress resistance of wild-type worms after 200 μM PA treatment, which revealed a significant increase (33.9% and 24.8%) in lifespan under heat stress (35 °C) and oxidative stress (20 mM paraquat), respectively. These findings suggest that PA has potential as an anti-aging and antioxidant drug.

Based on above lifespan experiment findings, it has been established that PA can increase the lifespan in C.elegans. Following, our research primarily focusesed on elucidating the mechanisms underlying the extension of nematode lifespan by PA. The insulin/insulin-like (IIS) growth factor signaling pathway is widely recognized as a key regulator of longevity, with the transcription factor DAF-16/FOXO playing a pivotal role in stress resistance and longevity regulation (Mendelski et al., 2019). In response to diverse environmental stimuli, insulin peptides released by the organism specifically bind to DAF-2, an IGF-1 receptor homolog that activates downstream signaling through the conserved PI3K/Akt pathway. This cascade commences with DAF-2 and ultimately influences FOXO/DAF-16, a transcription factor located downstream of the IIS pathway that transcribes genes associated with longevity upon nuclear entry (Blackwell et al., 2015). When environmental changes or genetic mutations impede IIS signaling, FOXO/DAF-16 remains unphosphorylated and translocates into the nucleus to activate senescence-related genes (e.g., ROS scavenging enzymes) (Miranda-Vizuete and Veal, 2017), thereby regulating senescence and longevity. Lowering IIS levels enhances adversity resistance and delays senescence in *C. elegans*; this effect is mediated by IIS inhibition of the downstream protein DAF-16. These findings suggest that the activation of DAF-16, along with its downstream genes, is essential for the PA-induced extension of lifespan, potentially involving modulation through the IIS pathway.

To ascertain whether PA exclusively regulates DAF-16 to prolong the lifespan of C.elegans via the IIS pathway, we investigated the IIS-mediated transcription factors HSF-1 and SKN-1.The results demonstrated that PA confers a certain level of resistance to heat stress and oxidative stress. However, none of these alterations were observed in the mutant strain.This finding is consistent with previous research indicating that DAF-16 interacts with other factors known to be implicated in the aging process (Zhu et al., 2020).

The relationship between mitochondrial damage and oxidative stress is tightly intertwined in *C. elegans*. (Tjahjono et al., 2020). Our findings indicate that PA treatment an upregulation of mitochondria-associated transcription factors mRNA levels following PA treatment in *C. elegans*. But it does not extend the lifespan of nematode mutants with impaired mitochondrial respiration. This suggests that PA may exert its effects on lifespan and oxidative stress extension protection through modulation of mitochondrial respiration. Lipid accumulation has been associated with various markers of oxidative stress (Yang et al., 2019). In wild-type nematodes, increased production of reactive oxygen species (ROS) may contribute to excessive lipid accumulation (Wang K. et al., 2018).

Therefore, we conducted oil red staining analysis in N2 nematodes treated with or without PA, to investigate the impact of PA on lipid accumulation. The results revealed a reduction in lipid accumulation in PA-treated nematodes. Additionally, we assessed the expression levels of target genes associated with lipid metabolism and observed a significant increase in mRNA expression levels following PA treatment.This suggests that PA may regulate oxidative stress and thus affect lipid metabolism in *C. elegans*.

Recent studies have revealed that autophagy gene cascades act downstream of IIS, targeting the target of the rapamycin (TOR) signaling pathway as well as mTOR and its downstream effector S6K, which play pivotal roles in numerous life extension interventions (Na. et al., 2017). Our results indicate that PA promotes autophagy. Furthermore, in atg-18 and bec-1 mutant nematodes treated with PA, the longevity phenotype disappeared, providing evidence for the association between PA-induced promotion of autophagy and autophagy-related genes. Furthermore, to establish a link between PA-induced autophagy and the mTOR signaling pathway, we evaluated the protein level of S6K, an important downstream gene regulated by mTOR. Remarkably, our findings showed a significant decrease in S6K protein level following PA treatment in *C. elegans*. These observations suggest that the lifespan extension induced by PA may be attributed to enhanced autophagy through activation of the mTOR pathway.The findings presented here are in line with those documented in the existing literature.

## 5 Conclusion

Based on the experimental findings, we hypothesize that PA potentially activates the transcription factors FOXO/DAF-16, HSF-1, and SKN-1 primarily through modulation of the insulin signaling pathway. Additionally, it is suggested that the effects of PA are contingent upon autophagy pathway regulation and the restoration of mitochondrial function. These mechanisms collectively contribute to enhanced stress resistance and an extended healthy lifespan in *C. elegans*. Given the limited research on the role of PAs in aging, our results suggest that PAs are promising candidates for the development of anti-aging drugs. However, it is important to note that these observations were limited to *C. elegans* as a model organism, and further investigations are warranted to validate these effects in other model organisms, such as mice. Additionally, further investigation into the pharmacological mechanisms and pharmaceutical applications of PAs is warranted.

## Data Availability

The original contributions presented in the study are included in the article/Supplementary material, further inquiries can be directed to the corresponding authors.
